# Supplementary material for: Hot spot prediction in protein-protein interactions by an ensemble system
Source: BMC Syst Biol. 2018 Dec 31;12(Suppl 9):132. doi: 10.1186/s12918-018-0665-8 (PMC6311905; doi:10.1186/s12918-018-0665-8)
Supplement: Supplementary file 2 — All relevant MATLAB code. A simple Matlab implement of our predictor. (ZIP 19,742 kb) [file 12918_2018_665_MOESM2_ESM.zip › Additional file 2/Read ASA/ASA website/dbMPIKT/1JTDB.html]

 NetSurfP - Protein Surface Accessibility and Secondary Structure Predictions

  

|  |  |  |
| --- | --- | --- |
|  |  | NetSurfP - Protein Surface Accessibility and Secondary Structure Predictions  Technical University of Denmark |

  


---

```
# For publication of results, please cite:
# A generic method for assignment of reliability scores applied to solvent accessibility predictions.
# Bent Petersen, Thomas Nordahl Petersen, Pernille Andersen, Morten Nielsen and Claus Lundegaard
# BMC Structural Biology 2009, 9:51 doi:10.1186/1472-6807-9-51
#
# Column 1: Class assignment - B for buried or E for Exposed - Threshold: 25% exposure, but not based on RSA
# Column 2: Amino acid
# Column 3: Sequence name
# Column 4: Amino acid number
# Column 5: Relative Surface Accessibility - RSA
# Column 6: Absolute Surface Accessibility
# Column 7: Z-fit score for RSA prediction
# Column 8: Probability for Alpha-Helix
# Column 9: Probability for Beta-strand
# Column 10: Probability for Coil
E V  1JTDB                  1    0.664 102.088  -1.080   0.003   0.003   0.994
E A  1JTDB                  2    0.563  61.999  -0.751   0.004   0.085   0.910
E A  1JTDB                  3    0.575  63.409   0.612   0.005   0.045   0.951
B T  1JTDB                  4    0.248  34.467  -1.440   0.004   0.138   0.858
E S  1JTDB                  5    0.333  39.074   1.747   0.002   0.816   0.182
B V  1JTDB                  6    0.038   5.794   0.619   0.001   0.959   0.040
B V  1JTDB                  7    0.093  14.294   1.592   0.000   0.983   0.017
B A  1JTDB                  8    0.026   2.898   0.392   0.001   0.959   0.040
B W  1JTDB                  9    0.044  10.486   0.723   0.001   0.900   0.099
B G  1JTDB                 10    0.036   2.794  -2.085   0.004   0.616   0.381
B G  1JTDB                 11    0.231  18.211  -0.398   0.004   0.197   0.799
B N  1JTDB                 12    0.175  25.620  -1.962   0.018   0.047   0.935
E N  1JTDB                 13    0.486  71.121  -1.757   0.018   0.019   0.964
E D  1JTDB                 14    0.468  67.511  -0.805   0.018   0.047   0.935
E W  1JTDB                 15    0.314  75.565  -1.773   0.018   0.088   0.893
E G  1JTDB                 16    0.278  21.871  -1.675   0.019   0.141   0.840
E E  1JTDB                 17    0.453  79.209  -1.352   0.021   0.279   0.699
B A  1JTDB                 18    0.187  20.640  -0.838   0.022   0.359   0.619
E T  1JTDB                 19    0.405  56.132  -1.139   0.022   0.359   0.619
B V  1JTDB                 20    0.166  25.499  -0.028   0.022   0.359   0.619
E P  1JTDB                 21    0.467  66.239  -0.743   0.005   0.262   0.733
B A  1JTDB                 22    0.199  21.930  -1.291   0.019   0.141   0.840
E E  1JTDB                 23    0.607 106.008  -1.575   0.018   0.047   0.935
E A  1JTDB                 24    0.411  45.248  -1.464   0.018   0.019   0.964
E Q  1JTDB                 25    0.586 104.713  -1.568   0.018   0.019   0.964
E S  1JTDB                 26    0.468  54.814  -0.730   0.018   0.019   0.964
E G  1JTDB                 27    0.467  36.784   1.330   0.004   0.085   0.910
B V  1JTDB                 28    0.046   7.101   0.371   0.003   0.718   0.279
E D  1JTDB                 29    0.237  34.209   1.129   0.001   0.900   0.099
B A  1JTDB                 30    0.210  23.131   1.040   0.001   0.959   0.040
B I  1JTDB                 31    0.023   4.218   1.045   0.001   0.959   0.040
B A  1JTDB                 32    0.054   5.951  -0.619   0.001   0.900   0.099
B G  1JTDB                 33    0.046   3.644  -0.509   0.004   0.616   0.381
B G  1JTDB                 34    0.100   7.839  -1.229   0.005   0.045   0.951
E Y  1JTDB                 35    0.358  76.590  -0.526   0.003   0.003   0.994
B F  1JTDB                 36    0.262  52.644  -0.323   0.004   0.138   0.858
B H  1JTDB                 37    0.037   6.748   0.429   0.002   0.816   0.182
B G  1JTDB                 38    0.029   2.314   0.184   0.001   0.959   0.040
B L  1JTDB                 39    0.021   3.845   1.342   0.001   0.959   0.040
B A  1JTDB                 40    0.021   2.303   1.074   0.001   0.959   0.040
B L  1JTDB                 41    0.035   6.409   0.762   0.001   0.959   0.040
B K  1JTDB                 42    0.183  37.725   0.333   0.002   0.816   0.182
E G  1JTDB                 43    0.529  41.624   0.109   0.005   0.045   0.951
E G  1JTDB                 44    0.352  27.671  -0.949   0.003   0.003   0.994
E K  1JTDB                 45    0.308  63.376   1.270   0.004   0.420   0.576
B V  1JTDB                 46    0.018   2.751   1.226   0.001   0.900   0.099
B L  1JTDB                 47    0.067  12.176   0.841   0.006   0.962   0.032
B G  1JTDB                 48    0.022   1.755   0.209   0.011   0.918   0.071
B W  1JTDB                 49    0.033   7.961   0.565   0.001   0.900   0.099
B G  1JTDB                 50    0.038   2.951  -2.373   0.004   0.616   0.381
B A  1JTDB                 51    0.248  27.341  -0.320   0.004   0.138   0.858
B N  1JTDB                 52    0.142  20.760  -1.606   0.005   0.015   0.979
E L  1JTDB                 53    0.588 107.608  -1.039   0.005   0.015   0.979
E N  1JTDB                 54    0.454  66.407  -1.234   0.005   0.015   0.979
B G  1JTDB                 55    0.138  10.821  -2.445   0.005   0.015   0.979
B Q  1JTDB                 56    0.150  26.736  -2.410   0.018   0.088   0.893
B L  1JTDB                 57    0.098  17.871  -1.088   0.019   0.141   0.840
B T  1JTDB                 58    0.172  23.926  -3.119   0.018   0.088   0.893
E M  1JTDB                 59    0.311  62.191  -0.041   0.018   0.047   0.935
E P  1JTDB                 60    0.361  51.297  -1.771   0.018   0.047   0.935
E A  1JTDB                 61    0.448  49.370  -1.421   0.018   0.047   0.935
E A  1JTDB                 62    0.490  53.954  -1.367   0.018   0.047   0.935
E T  1JTDB                 63    0.460  63.788  -1.112   0.018   0.047   0.935
E Q  1JTDB                 64    0.650 116.019  -1.112   0.018   0.019   0.964
E S  1JTDB                 65    0.421  49.329  -0.379   0.018   0.047   0.935
E G  1JTDB                 66    0.416  32.763   1.157   0.004   0.197   0.799
B V  1JTDB                 67    0.043   6.563   0.287   0.003   0.718   0.279
E D  1JTDB                 68    0.269  38.821   1.012   0.001   0.900   0.099
B A  1JTDB                 69    0.212  23.340   0.515   0.001   0.959   0.040
B I  1JTDB                 70    0.028   5.198   0.214   0.001   0.959   0.040
B A  1JTDB                 71    0.115  12.684  -0.495   0.002   0.816   0.182
B A  1JTDB                 72    0.046   5.080  -0.819   0.004   0.616   0.381
B G  1JTDB                 73    0.072   5.651  -1.342   0.004   0.085   0.910
E N  1JTDB                 74    0.381  55.778  -0.320   0.005   0.015   0.979
B Y  1JTDB                 75    0.249  53.254  -1.173   0.004   0.138   0.858
B H  1JTDB                 76    0.042   7.603   0.074   0.004   0.514   0.481
B S  1JTDB                 77    0.041   4.770  -1.055   0.002   0.816   0.182
B L  1JTDB                 78    0.025   4.651   0.726   0.001   0.959   0.040
B A  1JTDB                 79    0.081   8.882   0.174   0.001   0.959   0.040
B L  1JTDB                 80    0.050   9.082   0.331   0.001   0.900   0.099
B K  1JTDB                 81    0.217  44.657  -0.198   0.004   0.616   0.381
E D  1JTDB                 82    0.583  83.981  -0.648   0.005   0.045   0.951
E G  1JTDB                 83    0.450  35.454  -1.795   0.005   0.015   0.979
E E  1JTDB                 84    0.274  47.868   0.993   0.004   0.514   0.481
B V  1JTDB                 85    0.031   4.780  -0.233   0.001   0.900   0.099
B I  1JTDB                 86    0.084  15.577   1.162   0.011   0.918   0.071
B A  1JTDB                 87    0.025   2.766  -0.499   0.011   0.918   0.071
B W  1JTDB                 88    0.038   9.091  -0.130   0.018   0.846   0.136
B G  1JTDB                 89    0.043   3.408  -3.841   0.004   0.420   0.576
E G  1JTDB                 90    0.308  24.208  -0.777   0.004   0.138   0.858
B N  1JTDB                 91    0.279  40.875  -2.390   0.005   0.015   0.979
E E  1JTDB                 92    0.502  87.734  -1.774   0.018   0.047   0.935
E D  1JTDB                 93    0.488  70.321  -1.214   0.018   0.047   0.935
B G  1JTDB                 94    0.213  16.724  -2.577   0.018   0.047   0.935
E Q  1JTDB                 95    0.297  53.044  -2.088   0.018   0.088   0.893
B T  1JTDB                 96    0.127  17.657  -0.846   0.020   0.205   0.775
B T  1JTDB                 97    0.224  31.013  -2.569   0.020   0.205   0.775
B V  1JTDB                 98    0.248  38.164  -0.534   0.019   0.141   0.840
E P  1JTDB                 99    0.391  55.426  -1.705   0.018   0.047   0.935
B A  1JTDB                100    0.300  33.093  -1.588   0.018   0.047   0.935
E E  1JTDB                101    0.548  95.701  -1.859   0.018   0.019   0.964
E A  1JTDB                102    0.479  52.797  -1.975   0.018   0.019   0.964
E R  1JTDB                103    0.680 155.674  -1.825   0.018   0.019   0.964
E S  1JTDB                104    0.395  46.271  -0.750   0.005   0.045   0.951
E G  1JTDB                105    0.495  38.988   0.644   0.005   0.262   0.733
B V  1JTDB                106    0.059   9.114   0.622   0.003   0.718   0.279
E D  1JTDB                107    0.273  39.354   0.766   0.001   0.900   0.099
B A  1JTDB                108    0.239  26.305   0.408   0.001   0.959   0.040
B I  1JTDB                109    0.030   5.606  -0.126   0.001   0.900   0.099
B A  1JTDB                110    0.233  25.644  -0.082   0.001   0.900   0.099
B A  1JTDB                111    0.065   7.152  -0.992   0.004   0.616   0.381
E G  1JTDB                112    0.250  19.675  -1.583   0.004   0.085   0.910
E A  1JTDB                113    0.395  43.540  -1.236   0.005   0.015   0.979
E W  1JTDB                114    0.334  80.303  -0.785   0.004   0.197   0.799
B A  1JTDB                115    0.043   4.750   0.077   0.003   0.718   0.279
B S  1JTDB                116    0.043   4.981  -1.342   0.002   0.816   0.182
B Y  1JTDB                117    0.031   6.710   0.179   0.001   0.900   0.099
B A  1JTDB                118    0.025   2.711  -0.469   0.001   0.900   0.099
B L  1JTDB                119    0.049   8.899   0.160   0.001   0.900   0.099
B K  1JTDB                120    0.241  49.533  -0.390   0.003   0.718   0.279
E D  1JTDB                121    0.494  71.185  -0.771   0.005   0.045   0.951
E G  1JTDB                122    0.400  31.449  -1.962   0.003   0.003   0.994
E K  1JTDB                123    0.302  62.039  -0.332   0.005   0.336   0.660
B V  1JTDB                124    0.040   6.117  -0.851   0.002   0.816   0.182
B I  1JTDB                125    0.096  17.704   0.099   0.011   0.918   0.071
B A  1JTDB                126    0.032   3.493  -1.340   0.018   0.846   0.136
B W  1JTDB                127    0.059  14.286  -0.250   0.021   0.756   0.223
B G  1JTDB                128    0.092   7.256  -3.173   0.022   0.552   0.426
E D  1JTDB                129    0.321  46.271  -1.347   0.005   0.262   0.733
B D  1JTDB                130    0.212  30.506  -1.859   0.018   0.047   0.935
E S  1JTDB                131    0.596  69.839  -1.612   0.018   0.047   0.935
E D  1JTDB                132    0.523  75.350  -0.917   0.018   0.088   0.893
B G  1JTDB                133    0.252  19.825  -2.366   0.018   0.088   0.893
E Q  1JTDB                134    0.301  53.794  -1.684   0.021   0.279   0.699
B T  1JTDB                135    0.191  26.464  -0.911   0.021   0.451   0.528
B T  1JTDB                136    0.249  34.550  -2.394   0.022   0.359   0.619
B V  1JTDB                137    0.256  39.301  -0.167   0.020   0.205   0.775
E P  1JTDB                138    0.421  59.726  -1.497   0.019   0.141   0.840
E A  1JTDB                139    0.376  41.380  -1.896   0.018   0.088   0.893
E E  1JTDB                140    0.574 100.365  -1.532   0.018   0.088   0.893
E A  1JTDB                141    0.379  41.744  -1.621   0.018   0.019   0.964
E Q  1JTDB                142    0.559  99.802  -2.240   0.018   0.019   0.964
E S  1JTDB                143    0.523  61.249  -1.216   0.004   0.085   0.910
E G  1JTDB                144    0.451  35.517   0.030   0.004   0.420   0.576
B V  1JTDB                145    0.106  16.261   0.851   0.002   0.816   0.182
B T  1JTDB                146    0.238  33.024   0.203   0.001   0.900   0.099
B A  1JTDB                147    0.202  22.282   0.358   0.001   0.959   0.040
B L  1JTDB                148    0.031   5.694  -0.778   0.001   0.900   0.099
B D  1JTDB                149    0.066   9.439  -1.692   0.002   0.816   0.182
B G  1JTDB                150    0.064   5.037  -1.505   0.005   0.336   0.660
E G  1JTDB                151    0.226  17.826  -0.845   0.004   0.085   0.910
B V  1JTDB                152    0.262  40.269  -0.892   0.005   0.045   0.951
B Y  1JTDB                153    0.178  38.039  -0.429   0.005   0.336   0.660
B T  1JTDB                154    0.030   4.189  -0.108   0.002   0.816   0.182
B A  1JTDB                155    0.024   2.678   0.106   0.001   0.900   0.099
B L  1JTDB                156    0.025   4.504   0.779   0.001   0.959   0.040
B A  1JTDB                157    0.027   2.986   0.210   0.001   0.959   0.040
B V  1JTDB                158    0.084  12.849   0.104   0.001   0.900   0.099
E K  1JTDB                159    0.379  77.899   0.094   0.004   0.514   0.481
E N  1JTDB                160    0.625  91.456  -0.205   0.005   0.015   0.979
E G  1JTDB                161    0.432  33.959  -1.155   0.005   0.015   0.979
E G  1JTDB                162    0.299  23.516   1.075   0.004   0.616   0.381
B V  1JTDB                163    0.030   4.565   0.000   0.001   0.900   0.099
B I  1JTDB                164    0.079  14.634   0.989   0.006   0.962   0.032
B A  1JTDB                165    0.022   2.435   0.002   0.011   0.918   0.071
B W  1JTDB                166    0.042  10.101  -0.030   0.001   0.900   0.099
B G  1JTDB                167    0.061   4.840  -2.786   0.004   0.514   0.481
B D  1JTDB                168    0.287  41.429  -0.926   0.004   0.138   0.858
B N  1JTDB                169    0.215  31.432  -2.184   0.018   0.019   0.964
E Y  1JTDB                170    0.579 123.668  -1.934   0.018   0.047   0.935
E F  1JTDB                171    0.486  97.520  -1.988   0.018   0.047   0.935
E G  1JTDB                172    0.323  25.412  -1.993   0.018   0.047   0.935
E Q  1JTDB                173    0.393  70.261  -1.501   0.018   0.088   0.893
E T  1JTDB                174    0.330  45.757  -0.929   0.021   0.279   0.699
E T  1JTDB                175    0.358  49.668  -1.524   0.021   0.279   0.699
B V  1JTDB                176    0.190  29.203  -0.605   0.020   0.205   0.775
E P  1JTDB                177    0.482  68.367  -1.769   0.019   0.141   0.840
B A  1JTDB                178    0.299  32.895  -1.446   0.053   0.043   0.903
E E  1JTDB                179    0.520  90.896  -1.728   0.058   0.017   0.925
E A  1JTDB                180    0.448  49.348  -1.372   0.053   0.005   0.942
E Q  1JTDB                181    0.577 102.963  -1.937   0.018   0.019   0.964
E S  1JTDB                182    0.451  52.845  -0.738   0.018   0.019   0.964
E G  1JTDB                183    0.421  33.109   0.897   0.004   0.085   0.910
B V  1JTDB                184    0.041   6.379   0.557   0.004   0.616   0.381
B D  1JTDB                185    0.196  28.172   0.528   0.001   0.900   0.099
B D  1JTDB                186    0.167  24.079   0.545   0.001   0.959   0.040
B V  1JTDB                187    0.031   4.842  -0.127   0.001   0.900   0.099
B A  1JTDB                188    0.073   8.012  -0.800   0.001   0.900   0.099
B G  1JTDB                189    0.073   5.761  -0.823   0.004   0.514   0.481
B G  1JTDB                190    0.124   9.759  -1.749   0.004   0.085   0.910
E I  1JTDB                191    0.287  53.114  -0.925   0.005   0.045   0.951
B F  1JTDB                192    0.215  43.130  -0.798   0.005   0.336   0.660
B H  1JTDB                193    0.054   9.823   0.372   0.004   0.616   0.381
B S  1JTDB                194    0.040   4.700  -0.331   0.018   0.846   0.136
B L  1JTDB                195    0.026   4.797   0.782   0.011   0.918   0.071
B A  1JTDB                196    0.026   2.865   0.239   0.001   0.900   0.099
B L  1JTDB                197    0.053   9.686   0.714   0.001   0.959   0.040
B K  1JTDB                198    0.226  46.468  -0.103   0.002   0.816   0.182
E D  1JTDB                199    0.510  73.534  -0.290   0.004   0.085   0.910
E G  1JTDB                200    0.381  30.024  -0.965   0.005   0.015   0.979
E K  1JTDB                201    0.272  55.992   1.249   0.004   0.616   0.381
B V  1JTDB                202    0.019   2.843   1.129   0.001   0.959   0.040
B I  1JTDB                203    0.063  11.655   1.186   0.001   0.959   0.040
B A  1JTDB                204    0.019   2.127   0.623   0.001   0.959   0.040
B W  1JTDB                205    0.032   7.792   0.410   0.001   0.900   0.099
B G  1JTDB                206    0.028   2.188  -1.897   0.003   0.718   0.279
B D  1JTDB                207    0.236  34.008  -0.405   0.005   0.262   0.733
B N  1JTDB                208    0.146  21.418  -2.348   0.005   0.045   0.951
E R  1JTDB                209    0.571 130.667  -1.141   0.018   0.019   0.964
E Y  1JTDB                210    0.452  96.550  -1.613   0.018   0.019   0.964
B K  1JTDB                211    0.263  54.017  -1.680   0.018   0.047   0.935
B Q  1JTDB                212    0.242  43.239  -3.051   0.019   0.141   0.840
B T  1JTDB                213    0.171  23.704  -1.464   0.021   0.279   0.699
B T  1JTDB                214    0.251  34.855  -2.716   0.020   0.205   0.775
E V  1JTDB                215    0.344  52.950  -0.096   0.018   0.088   0.893
E P  1JTDB                216    0.388  55.000  -1.779   0.018   0.088   0.893
B T  1JTDB                217    0.273  37.921  -1.303   0.018   0.047   0.935
E E  1JTDB                218    0.638 111.511  -1.672   0.018   0.019   0.964
E A  1JTDB                219    0.497  54.736  -1.347   0.018   0.019   0.964
E L  1JTDB                220    0.392  71.702  -1.569   0.018   0.019   0.964
E S  1JTDB                221    0.476  55.846  -0.601   0.018   0.019   0.964
E G  1JTDB                222    0.431  33.928   1.261   0.004   0.138   0.858
B V  1JTDB                223    0.036   5.549   0.718   0.003   0.718   0.279
E S  1JTDB                224    0.243  28.491   1.018   0.001   0.900   0.099
B A  1JTDB                225    0.214  23.583   1.309   0.001   0.959   0.040
B I  1JTDB                226    0.022   4.144   1.004   0.001   0.959   0.040
B A  1JTDB                227    0.055   6.050  -0.607   0.001   0.959   0.040
B S  1JTDB                228    0.033   3.821  -0.776   0.003   0.718   0.279
B G  1JTDB                229    0.066   5.218  -0.548   0.005   0.045   0.951
E E  1JTDB                230    0.333  58.105  -0.682   0.003   0.003   0.994
B W  1JTDB                231    0.213  51.106  -0.710   0.004   0.138   0.858
B Y  1JTDB                232    0.036   7.693   0.199   0.003   0.718   0.279
B S  1JTDB                233    0.026   3.071   0.335   0.001   0.900   0.099
B L  1JTDB                234    0.017   3.094   1.603   0.001   0.959   0.040
B A  1JTDB                235    0.018   1.928   1.149   0.001   0.959   0.040
B L  1JTDB                236    0.047   8.606   0.773   0.001   0.959   0.040
B K  1JTDB                237    0.105  21.681   0.239   0.002   0.816   0.182
E N  1JTDB                238    0.445  65.192  -0.059   0.005   0.045   0.951
E G  1JTDB                239    0.393  30.953  -1.347   0.003   0.003   0.994
B K  1JTDB                240    0.245  50.335   1.130   0.005   0.336   0.660
B V  1JTDB                241    0.017   2.644   1.244   0.001   0.900   0.099
B I  1JTDB                242    0.059  10.823   1.079   0.006   0.962   0.032
B A  1JTDB                243    0.021   2.336   0.632   0.006   0.962   0.032
B W  1JTDB                244    0.029   7.047   0.690   0.001   0.900   0.099
B G  1JTDB                245    0.027   2.164  -1.882   0.004   0.616   0.381
B S  1JTDB                246    0.252  29.511  -0.061   0.004   0.138   0.858
B S  1JTDB                247    0.120  14.029  -1.987   0.005   0.015   0.979
E R  1JTDB                248    0.474 108.569  -0.669   0.005   0.015   0.979
E T  1JTDB                249    0.391  54.287  -1.117   0.005   0.045   0.951
B A  1JTDB                250    0.082   9.058  -1.495   0.005   0.045   0.951
B P  1JTDB                251    0.146  20.703  -1.711   0.018   0.088   0.893
B S  1JTDB                252    0.073   8.509  -1.435   0.018   0.088   0.893
B S  1JTDB                253    0.120  14.017  -3.064   0.018   0.088   0.893
E V  1JTDB                254    0.331  50.952  -0.157   0.018   0.019   0.964
E Q  1JTDB                255    0.394  70.315  -1.345   0.005   0.015   0.979
E S  1JTDB                256    0.646  75.664  -1.772   0.003   0.003   0.994
E G  1JTDB                257    0.605  47.614   0.169   0.005   0.015   0.979
B V  1JTDB                258    0.117  17.998  -0.073   0.004   0.197   0.799
E S  1JTDB                259    0.458  53.619   0.587   0.004   0.514   0.481
E S  1JTDB                260    0.315  36.895   0.583   0.002   0.816   0.182
B I  1JTDB                261    0.059  10.823  -0.348   0.001   0.900   0.099
B E  1JTDB                262    0.143  25.000  -0.712   0.003   0.718   0.279
B A  1JTDB                263    0.079   8.717  -0.928   0.005   0.336   0.660
B G  1JTDB                264    0.068   5.320  -0.519   0.004   0.085   0.910
E P  1JTDB                265    0.360  51.027  -0.849   0.018   0.019   0.964
B N  1JTDB                266    0.287  42.017  -0.740   0.005   0.045   0.951
B A  1JTDB                267    0.053   5.841   0.356   0.004   0.616   0.381
B A  1JTDB                268    0.033   3.692  -0.843   0.001   0.900   0.099
B Y  1JTDB                269    0.033   7.159   0.047   0.001   0.900   0.099
B A  1JTDB                270    0.028   3.086  -1.024   0.001   0.900   0.099
B L  1JTDB                271    0.050   9.063  -0.832   0.001   0.900   0.099
E K  1JTDB                272    0.358  73.579   1.247   0.003   0.718   0.279
E G  1JTDB                273    0.718  56.538  -2.552   0.003   0.003   0.994

Explain the output.  Go back.
```

---
